# Editorial: Gambling, stigma, suicidality, and the internalization of the ‘responsible gambling’ mantra

**DOI:** 10.3389/fpsyt.2023.1214531

**Published:** 2023-06-01

**Authors:** Angela Rintoul, Virve Marionneau, Charles Livingstone, Janne Nikkinen, Chebiwot Kipsaina

**Affiliations:** ^1^Health Innovation and Transformation Centre, Research and Innovation, Federation University, Churchill, VIC, Australia; ^2^Department of Epidemiology and Preventive Medicine, School of Public Health and Preventive Medicine, Monash University, Melbourne, VIC, Australia; ^3^Centre for Research on Addiction, Control, and Governance, University of Helsinki, Faculty of Social Sciences, Helsinki, Finland; ^4^Curtin University, Perth, WA, Australia

**Keywords:** gambling, suicide prevention, harm reduction, commercial determinants of health, discourse, framing, responsible gambling

Gambling has traditionally been framed by industry, governments and even some academic researchers as a source of recreation, entertainment, and fun (1, 2). In established markets where gambling has been legal for many decades, it has arguably been normalized (3). Yet the reality for those who gamble at high-risk levels belies notions of glamor and excitement that often accompany these popular gambling myths. Framings matter: they determine how gambling is perceived and regulated (2). If gambling is framed as entertainment rather than as a public health concern, regulation is unlikely to be effective in terms of preventing the many harms that gambling causes to individuals, families, and societies (4).

The dominant “responsible gambling” paradigm focuses on individual responsibility. It leads to suboptimal regulation that does not target commercial practices, harmful products, and normalization which are the root of gambling harms. If individuals are seen to bear the sole responsibility for their gambling, those who are unable to control themselves are seen as “irresponsible” and stigmatized (5). The stigma of excessive gambling is often internalized by the individuals who gamble. This can lead to concealing problems and avoiding treatment (6) which can further aggravate harm.

In recent years, research on the harms connected to gambling has become more established. This has occurred alongside concerns about the conflicted evidence base upon which regulation is based, and the ways in which vested (commercial) interests have distorted our understanding of the locus of harm production (7–9). Suicide is among the most severe harmful consequences of gambling, and evidence of a link between gambling, suicidality and suicide is rapidly mounting (10, 11). Systematic reviews have established that gambling is a risk factor for suicide (10) and recent longitudinal evidence suggests that besides clinical problem gambling, any increase in the measured severity of problem gambling is linked to increased suicidality among young adults (12).

The objective of this Research Topic is to further this understanding on the relationship between gambling, self-harm and suicide; to inform understanding of the relationship between industry-oriented discourses like “responsible gaming/gambling” and the “problem gambler”; and, to consider the consequences of the internalization of these discourses. The topic presents papers that explore these questions via a scoping review of gambling-related suicides, from the perspectives of people who experience harm from their own gambling in Sweden, active military personnel in the UK, affected others in Japan, and via the “peculiar” intervention of self-exclusion.

Samuelsson and Cisneros Örnberg provide a detailed analysis of the concept of responsibility from the perspective of those experiencing gambling harm in Sweden. They present their analysis through a prism of an incongruent tension between the responsibility of the individual gambler and their medical diagnosis that relies upon symptoms that include an inability to control their gambling behavior. Through interviews with people who gamble at high-risk levels, the authors describe the ways in which people with lived experience of gambling harm interpret the responsibilities of actors throughout the gambling system.

Kraus et al. describe the differing regulatory conditions for self-exclusion programs across seven jurisdictions. Like Samuelsson and Cisneros Örnberg, these authors identify a critical tension between the voluntary action required by an individual who is unable to act in line with their best interests. They reflect that this “peculiar” yet widely adopted measure has no comparison in analogous fields and conclude that this measure represents a violation of governments' responsibility to protect consumers from this harmful commodity.

Marionneau and Nikkinen outline the complexities in gambling-related suicide studies. They find that in almost all reported qualitative evidence, gambling has been a direct contributor to suicidality. Stress related to debt and shame are major factors that lead those who gamble to a state of suicidality or suicide, and these same factors create barriers to seeking support and treatment. Acknowledging that multiple stressors are experienced by those who reach this point of crisis, they highlight the need for further evidence of the temporal order of gambling harm and confounding stressors. They find that evidence of the most effective forms of treatment and support is limited, and that ultimately the most effective measures will be underpinned by a comprehensive public health approach, including a focus on reducing stigma from gambling.

Takiguchi et al. provide a rare insight into the unique regulatory context of gambling in Japan, a country in the process of legalizing casinos. While gambling has technically been illegal in Japan until now, the authors describe the inconsistencies with gambling practices including the ubiquitous *pachinko* and *pashinko slot* machines. They remind us that the burden of gambling harm extends to “affected others,” who describe the shame and blame they internalized as a result of their family members gambling. Through their findings, Takiguchi et al. demonstrate how the industry-oriented discourse of responsible gambling has led to harmful consequences for parents, partners, and children of people who experience gambling harm. This framing has been effective in obscuring the role of industry and government in the production of gambling-related harm.

Champion et al. report the unique experiences of active, serving military personnel who undertake risky work, typically in situations of serious stress. In their article “Gambling problems and help-seeking in serving United Kingdom military personnel: A qualitative study” they find that occupational hazards associated with military service, combined with organizational norms and practices make this population particularly vulnerable to gambling-related harm. They conclude that more research is needed to screen for and support those experiencing gambling harm.

This collection of articles reaffirms that those experiencing harm from their gambling have often been cast as flawed consumers of a mostly harmless recreational pastime. Such framings are likely to compound the sense of shame and contribute to stigma. This creates barriers to understanding the true extent of gambling harm, undermines and misguides regulation of harmful products, and critically, impedes access to treatment and support. We hope this Research Topic can contribute to a critical reframing of gambling policy, research, and practices.

## Author contributions

AR conceptualized the Research Topic and drafted the editorial. VM, CL, JN, and CK provided input and revisions and approved the content prior to submission.
